# Telomerase Activity and Telomere Length in *Daphnia*

**DOI:** 10.1371/journal.pone.0127196

**Published:** 2015-05-11

**Authors:** Charles Schumpert, Jacob Nelson, Eunsuk Kim, Jeffry L. Dudycha, Rekha C. Patel

**Affiliations:** Department of Biological Sciences, University of South Carolina, Columbia, South Carolina, United States of America; University of Newcastle, UNITED KINGDOM

## Abstract

Telomeres, comprised of short repetitive sequences, are essential for genome stability and have been studied in relation to cellular senescence and aging. Telomerase, the enzyme that adds telomeric repeats to chromosome ends, is essential for maintaining the overall telomere length. A lack of telomerase activity in mammalian somatic cells results in progressive shortening of telomeres with each cellular replication event. Mammals exhibit high rates of cell proliferation during embryonic and juvenile stages but very little somatic cell proliferation occurs during adult and senescent stages. The telomere hypothesis of cellular aging states that telomeres serve as an internal mitotic clock and telomere length erosion leads to cellular senescence and eventual cell death. In this report, we have examined telomerase activity, processivity, and telomere length in *Daphnia*, an organism that grows continuously throughout its life. Similar to insects, *Daphnia* telomeric repeat sequence was determined to be TTAGG and telomerase products with five-nucleotide periodicity were generated in the telomerase activity assay. We investigated telomerase function and telomere lengths in two closely related ecotypes of *Daphnia* with divergent lifespans, short-lived *D*. *pulex* and long-lived *D*. *pulicaria*. Our results indicate that there is no age-dependent decline in telomere length, telomerase activity, or processivity in short-lived *D*. *pulex*. On the contrary, a significant age dependent decline in telomere length, telomerase activity and processivity is observed during life span in long-lived *D*. *pulicaria*. While providing the first report on characterization of *Daphnia* telomeres and telomerase activity, our results also indicate that mechanisms other than telomere shortening may be responsible for the strikingly short life span of *D*. *pulex*.

## Introduction

Telomeres, the ends of linear chromosomes, have been studied extensively in relation to cellular aging and senescence [1,2,3]. Composed of repetitive nucleotide sequences (TTAGGG for mammals) associated with proteins, telomeres protect important genetic information of linear chromosomes from deletion arising due to the “end replication” problem [1,3]. The process of DNA replication leads to progressive shortening of linear chromosomes at the telomeres due to the fact that DNA polymerases can only polymerize in a 5’ to 3’ direction and require a primer with a free 3’-OH group [1,3]. This inability to replicate linear DNA on the lagging strands all the way to ends necessitates the telomerase, an enzyme responsible for *de novo* addition of telomeric repeats to chromosomal ends [4]. Telomerase is a ribonucleoprotein complex, comprised of a protein catalytic subunit TERT (Telomeric Reverse Transcriptase), and an RNA template termed TERC (telomeric RNA Component) [1,3]. Telomerase activity is essential for maintaining telomere length throughout cellular lifespan. Early in human development, telomerase is constitutively active in cells but after birth it is active predominately in stem cells and germ cells with most somatic tissues having no telomerase activity [5,6]. Each individual DNA replication event of human telomerase-negative somatic cells leads to a loss of 100 bp of telomeric sequence, resulting in a progressive decline in telomere length with each cellular division [7]. Because of this progressive telomere shortening, human somatic cells can only undergo approximately 50 to 80 cellular replication events before becoming senescent [7]. Thus, telomere length is essential for normal cellular function and proliferation as well as chromosome stability. In the absence of proper telomere complex formation, the double-stranded break repair pathway can be initiated resulting in apoptosis or senescence [8,9]. Thus, telomeres serve a protective molecular role by shielding the loss of important genetic information as well as by maintaining chromosome stability throughout the cellular lifespan. Telomerase has also been implicated in nuclear DNA damage repair and plays a protective role for mitochondrial DNA during oxidative stress response during which telomerase shuttles from the nucleus to the mitochondria [10,11,12,13,14,15].

In this study we investigated telomerase activity, the telomeric repeat sequence, and telomere lengths in *Daphnia*, a freshwater crustacean, and an emerging model in aging liuresearch. *Daphnia* has been used extensively as a model in ecotoxicology studies [16] and with a fully sequenced genome of *D*. *pulex*, it is an emerging model in biomedical research [17,18]. Two ecotypes of *Daphnia* are of interest in relation to aging, *D*. *pulex* and *D*. *pulicaria* [19,20,21,22,23,24]. *D*. *pulex* is found in small transitory ponds, in which selection favors short longevity due to the limited time the ponds have water. In a laboratory environment *D*. *pulex* exhibits a lifespan on average of about 20 days [20,23]. *D*. *pulicaria* lives primarily in a stable environment of stratified lakes that are present all year long. In the lab *D*. *pulicaria* exhibits lifespans on average of about 70 days [20,23]. Genetically, the two ecotypes are almost identical and are capable of interbreeding with viable offspring in the wild [21]. *Daphnia* can be easily cultured and undergo cyclic parthenogenduesis [16], thus enabling creation of clonal lineages without the genetic variation normally associated with sexually reproducing organisms. Being crustaceans, *Daphnia* constantly shed their outer carapace and have regenerative cellular capacities [16]. Due to these unique characteristics *Daphnia* is a interesting model organism for understanding cellular processes associated with of aging.

We present characterization of the telomere length, telomerase activity and processivity in the two ecotypes, the short-lived *D*. *pulex* and the long lived *D*. *pulicaria*. In the short-lived *D*. *pulex*, telomere length did not decline with age; however, in the long-lived *D*. *pulicaria*, telomere length decreased with age. Accordingly, telomerase activity in *D*. *pulex* is relatively constant throughout the life span, whereas in *D*. *pulicaria*, it declines considerably with age. In addition, the telomerase processivity increased with age in *D*. *pulex*, whereas in *D*. *pulicaria* it declined with age. This is an important initial study to investigate telomere length and telomerase activity in a newly emerging model system for research on aging.

## Materials and Methods

### *Daphnia* Cultures

*Daphnia pulex* (clone RW 20) and *Daphnia pulicaria* (clone Lake XVI-11) were isolated from populations in southwest Michigan in 2008 and have since been cultured in the lab. No specific permissions are required to collect zooplankton from these public-access waterbodies in Michigan. *D*. *pulex* and *D*. *pulicara* are neither endagered nor protected. For further details on the source populations, see Dudycha [21]. *Daphnia* were maintained at 20°C with a photoperiod of 12:12 L:D (12 hours of light followed by 12 hours of dark) within a Percival growth chamber. *Daphnia* were maintained at 3 to 5 animals per 250 mL beaker in 200 mL of filtered lake water. *Daphnia* were cleared of young and transferred to a new beaker with fresh water on alternate days. They were fed every day with vitamin supplemented algae *Ankistrodesmus falcatus* at a concentration of 20,000 cells/mL. To generate experimental animals, even-aged cohorts were obtained by placing neonates individually in 100 mL of hardwater COMBO, or artificial lake water. Experimental animals were otherwise maintained as in the source cultures.

All *Daphnia* were maintained clonally in the lab and all *Daphnia* used for experimentation are diploid females. Once individuals were collected from the field, they were placed in beakers of fresh lake water and allowed to clonally reproduce. In this manner, each isolate, or clone, of *Daphnia* was produced (for example, individuals from the pond named Rough Wood were placed individually into beakers and designated as clone RW followed by a number which corresponds to the original analysis of the collected *Daphnia*). After each clone was established, *Daphnia* were maintained by constant feeding of algae and changing of the lake water to create an isogenic clonal line of *Daphnia*. Care was taken not to induce stress for the *Daphnia* such that sexual reproduction would not occur in the clonal lines.

### Telomeric Repeat Amplification Protocol (TRAP) Assay

Telomeric repeat amplification protocol (TRAP) Assay was performed using the TRAPeze Telomerase Detection kit (Millipore). *D*. *pulex* and *D*. *pulicaria* were collected at different ages. For *D*. *pulex* (RW20), we used 1, 2, and 3 week old individuals and for *D*. *pulicaria* (LakeXVI-11) we used 1, 4, and 8 week old individuals. To prepare cell extracts from *D*. *pulex* and *D*. *pulicaria*, 25 to 30 individual *Daphnia* were drained of all residual water, and homogenized in 200 μl of 1X CHAPS Lysis Buffer on ice. The homogenate was incubated on ice for 30 min, centrifuged at 12000 X g for 20 min at 4°C, 160 μl of extract was transferred to a new tube, and the total protein concentration in the extract was determined using a Bradford Assay. Samples were stored at -80°C.

The TRAP Assay was performed as per the instructions in the TRAPeze kit. A 50 μl reaction was performed for all samples and controls. A positive control of human cancer cell line extract was provided with the kit. Each reaction contained 5 μl of 10X TRAP reaction buffer, 1 μl of 50X dNTP mix, 1 μl of TS Primer, 1 μl of TRAP primer mix, 0.4 microliters (2 units) of Taq polymerase, 39.6 μl of deionized water, and 2 μl of extract (either control or sample). Tubes containing each reaction were placed in the thermocycler and heated to 30°C for 30 min. Then 30–33 cycles of 94°C for 30 sec, 59°C for 30 sec, and 72°C for 1 min were performed. The heat-inactivated control samples, were incubated at 85°C for 10 min prior to TRAP assay.

Following PCR amplification, 5 μl of loading dye (with bromophenol blue (0.25% in 50% glycerol/50 mM EDTA) and xylene cyanol (0.25% in 50% glycerol/50 mM EDTA) was mixed with the TRAPeze product. 25 μl was loaded into a 10% non-denaturing polyacrylamide gel and the gel was run at 400 V for 1.5 hours in 0.5 X TBE buffer, stained using Sybr Green for 30 min before scanning in a Typhoon FLA 7000.

### Quantification of the TRAP Assay Results

TRAP Assay results were quantified using a formula described in TRAPeze kit manual. The formula takes into account the signal measured in each reaction/lane (x) as well as the heat inactivated control (x_O_), the no-template control (r_O_), and the TSR8 quantitation control (r) reactions/lanes. All of these reactions contain an internal standard (36 bp band) engineered into the assay, which was also used in the formula (c for samples, c_r_ for TSR8 control). The resulting quantification was in units of Total Product Generated (TPG), which corresponds to the number of TS primers extended by at least 4 telomeric repeats in 30 min at 30°C.

$$TPG\;=\;\frac{(x-xo)/c}{(r-ro)/cr}\;x\;100$$

### Sequencing of the TRAP Product

A TRAP Assay reaction that contained a strong positive result from the *Daphnia* samples was used to determine the telomeric repeat sequence for *Daphnia*. 5 μl of the reaction products was purified using a Qiagen PCR Purification kit, and ligated into the vector pGEMT Easy (Promega), and sequenced.

### Survivorship studies

We compared survivorship of the XVI-11 and RW20 clones using standard life table methods for *Daphnia* [25]. Experimental conditions were 20°C, a 12:12 L:D photoperiod, with animals kept in individual 150-ml pyrex beakers in 100 ml COMBO hardwater artificial lakewater [26]. Two generations were maintained under experimental conditions prior to initiating the life table to minimize maternal effects variation. Experimental individuals were fed 20,000 cells/ml *Ankistrodesmus falcatus* daily, a food level that produces normal aging processes in *Daphnia* [20]. Individuals were transfered to fresh beakers and COMBO every other day, and survivorship was observed daily until all experimental animals died. For each clone, *n* = 60 females.

### Terminal Restriction Fragment (TRF) Assay

We preformed a Terminal Restriction Fragment Assay using a previously established protocol by Herbert *et al*. [27]. Genomic DNA was extracted from *D*. *pulex* and *D*. *pulicaria* at various ages using a CTAB (Hexadecyltrimethylammonium Bromide) based protocol optimized for use with *Daphnia*. For each aged set of *Daphnia*, 25 individuals were pooled for the extraction of genomic DNA. Each individual *Daphnia* was cleared of all embryos before the genomic DNA was extracted to ensure the results were reflective of the adult telomere length. One μg of genomic DNA was digested at 37°C overnight with 1 U/μl of the following restriction enzymes: HinfI, RsaI, MspI, CfoI, HaeII and AluI. Digests were run on a 0.7% agarose gel. Samples were run for 4 h at 120 V in 1X TAE Buffer to achieve desirable separation in size range 1 kb- 25 kb. The gel was denatured for 20 min in denaturing solution (0.5 M NaOH, 1.5M NaCl), and rinsed in distilled water for 10 min. The gel was then dried upside down between 2 sheets of Whatman 3MM filter paper under vacuum for 1 hour at 50°C. After removing the gel from the dryer, the gel was neutralized for 15 min in neutralizing solution (1.5 M NaCl, 0.5 M Tris-Cl, pH 8.0) and then rinsed with distilled water for 10 min. The gel was then soaked in 10 ml of prehybridization buffer (5X SSC Buffer, 5X Denhardt solution, 10 mM Na_2_HPO_4_, 1 mM Na_2_H_2_P_2_O_7_) for 10 min. The gel was then transferred to hybridization solution (5X SSC Buffer, 5X Denhardt solution, 10 mM Na_2_HPO_4_, 1 mM Na_2_H_2_P_2_O_7_) containing the radiolabeled telomeric sequence probe (see below). The gel was hybridized overnight at 42°C. After hybridization, the gel was washed once in 2X SSC for 15 min at 22°C, washed four times for 10 min each in 0.1X SSC/0.1% SDS. Following washes, the gel was exposed to a phosphor screen overnight and scanned on a Typhoon FLA 7000 phosphor imager and visualized with ImageQuant software.

### *Daphnia* telomeric probe design and labeling

Based on the telomeric repeat sequence for *Daphnia*, a telomeric probe was designed as 6 repeats of the telomeric repeat: 5’-TTAGGTTAGGTTAGGTTAGGTTAGGTTAGG-3’. The probe was 5’ end-labeled with γ-P^32^ ATP using polynucleotide kinase. To label the probe, 2 μl of 20 pmol/μl of oligonucleotide, 24 μl of γ-P^32^ ATP (3000 Ci/mmol), 10 μl 5X forward reaction buffer, 2 μl 10 U/μl T4 polynucleotide kinase, and 12 μl H_2_0 were added to the reaction mix and incubated at 37°C for 30 min. A QIAquick nucleotide removal kit was used to remove the unincorporated radioactivity. The labeling and specific activity of probe was determined by counting total cpm in a 1 μl aliquot (total 200 μl) using a scintillation counter.

### Statistics

To determine statistical significance of results of the TRAP assay, as well as TRF Assay, a two-tailed Student’s T-test was performed, assuming equal variance. Each figure legend denotes p values as denoted by brackets and special characters. Note that our alpha level was p = 0.05. A nonparametric log-rank test was performed for significance of the survival curves.

## Results

### Telomerase activity is present in *Daphnia* samples

**Fig 1:**
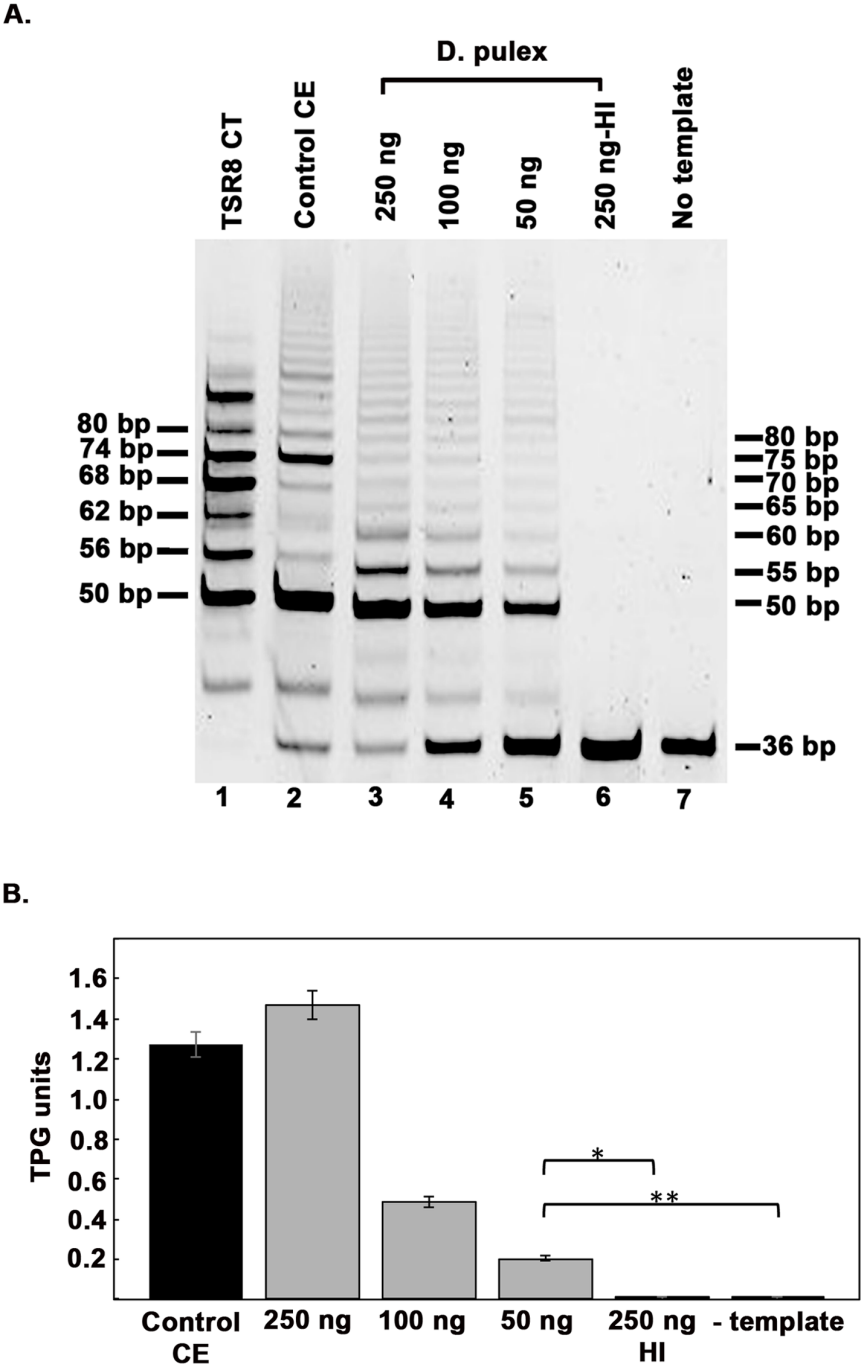
Telomerase activity in Daphnia. A) Telomeric repeat amplification protocol (TRAP) assay of *D*. *pulex* extracts. The amount of cell extract used is indicated above each lane. HI: Heat Inactivated, CE: Cell Extract. TSR8 CT: positive control for PCR step. B) Quantification of the TRAP Assay in 1A. Student T-test was performed, p values are as follows: * = 6.15x10^-6^, ** = 6.63x10^-6^ (n = 3).

To detect and assay for telomerase activity from adult *Daphnia*, we performed a telomerase repeat amplification protocol (TRAP) assay, using the TRAPeze (Millipore) kit. The kit is optimized for human telomerase activity, thus we tested it for use with *Daphnia* extracts. As represented in Fig 1A, we could detect strong telomerase activity in extracts prepared from adult *Daphnia* (lanes 3–5). Telomerase activity showed dose-dependence with amount of total protein in extracts. Each TRAP reaction mixture contains primers as well as a template for amplification of a 36 bp internal control, which also serves to determine false negative results due to the presence of a telomerase inhibitor in the extracts. *Daphnia* extracts did not show any inhibition of the telomerase activity compared to the positive control (lane 2) provided with the kit. The dependence of the band ladder on telomerase activity was confirmed by heat treatment of the *Daphnia* extract (lane 6), which completely eliminated the ladder due to inactivation of telomerase, thus confirming the presence of telomerase activity in *Daphnia* extract. Absence of bands with no template added (lane 7), indicated no contamination in the PCR step of the assay and further confirmed the presence of telomerase activity in *Daphnia* extract. The periodicity of the bands generated using *Daphnia* extract (lanes 3–5) was different than the periodicity obtained with extract from telomerase positive human cell line HEK293 (positive control- lane 1). Fig 1B represents quantification of telomerase activity.

**Fig 2:**
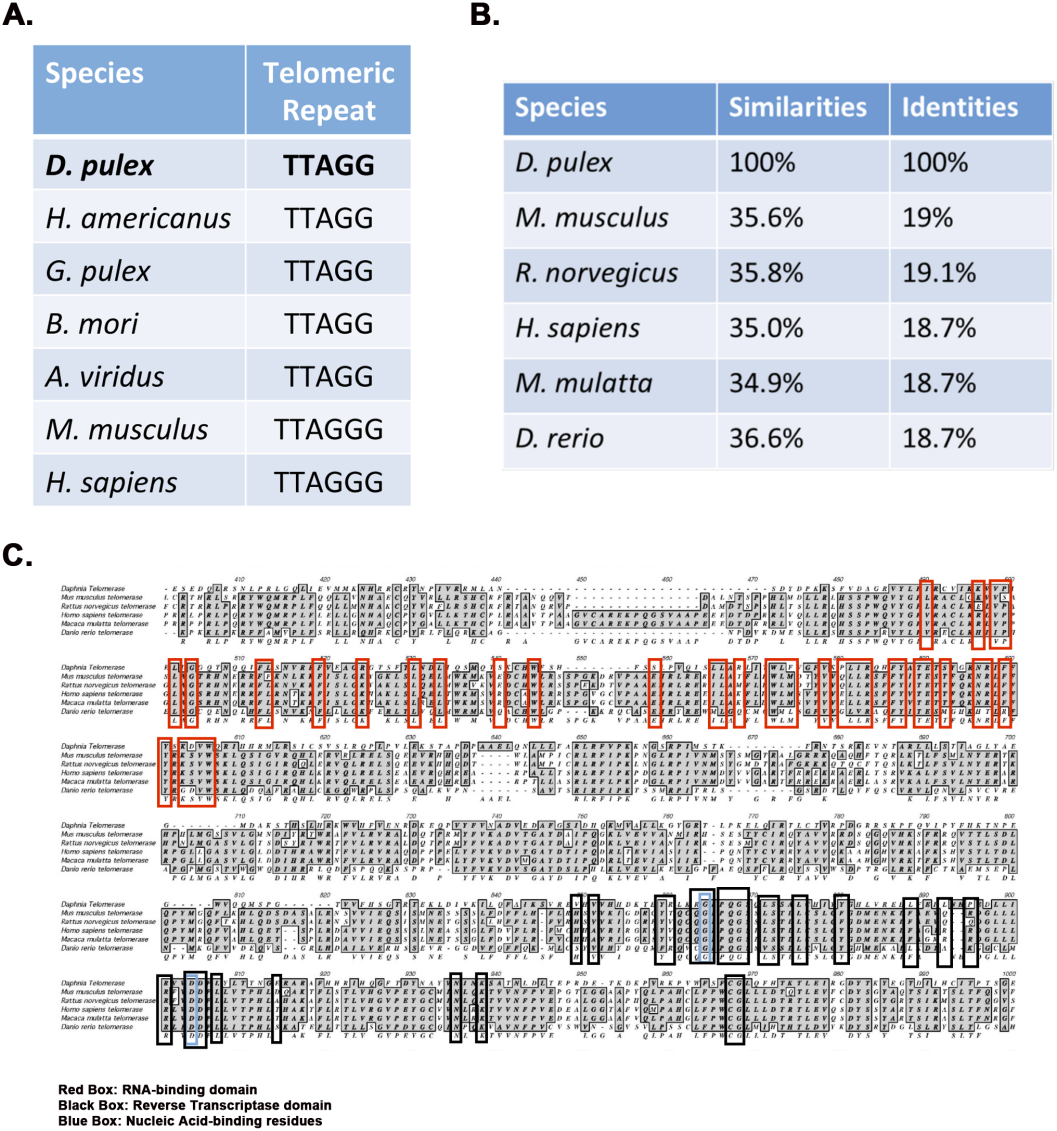
Telomeric repeat sequence from *Daphnia* and sequence alignment of *Daphnia* telomerase (dTERT) with telomerase proteins from other species. A) Telomeric repeat sequence of *Daphnia* and other species. *H*. *americanus*: *Homarus americanus*—Lobster, *G*. *pulex*: *Gammarus pulex*—Freshwater amphipod, *B*. *mori*: *Bombyx mori—*Silkworm/moth, *A*. *viridus*: *Amaranthus viridus*—Beetle, *M*. *musculus*: *Mus musculus*—Mouse, *H*. *sapiens*: *Homo sapiens*—Human. B) Identity and similarity percentages of dTERT with TERT from other species. C) *D*. *pulex* telomerase reverse transcriptase (DTERT) sequence alignment for the RNA-binding and reverse transcriptase domains. Green Boxes: conserved residues within the RNA binding domain. Black Boxes: conserved residues within the reverse transcriptase domain. Blue boxes: residues within the reverse transcriptase domain that are essential for nucleic acid binding.

Human telomeres have a six-nucleotide telomeric repeat sequence (TTAGGG). To determine the telomeric repeat length and sequence in *Daphnia*, we cloned and sequenced the *Daphnia* TRAP reaction products. As seen in Fig 2A, the sequence analysis of the clones revealed that the telomeric repeat was TTAGG, a sequence identical to crustaceans *H*. *americanus and G*. *pulex* [28]. The five nucleotide repeat sequence corresponds to the observed difference in the periodicity of bands in TRAP assay (Fig 1A, lanes 3–5), as the bands are expected to be shorter by one nucleotide as compared to TRAP assay products obtained with human cell extract (Fig 1A, lane 2). When telomerase (TERT) sequences across various species were compared, the similarity between the *D*. *pulex* TERT sequence and those of other organisms is relatively low for the entire protein (Fig 2B). The highest similarity is found in the essential functional domains of the protein: the RNA binding domain and the reverse transcriptase domain (Fig 2C), with high degree of sequence conservation.

### Telomerase in *Daphnia* embryos is more processive than in adult organisms

**Fig 3:**
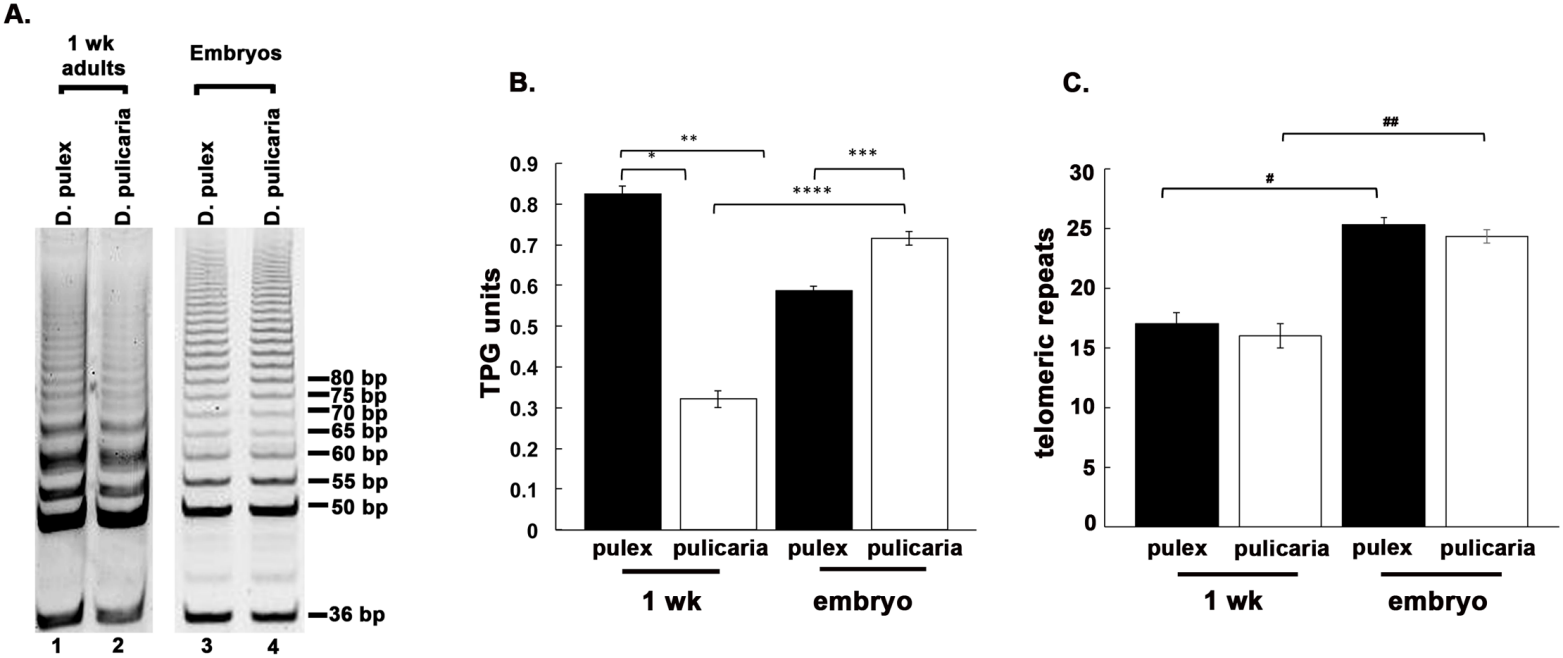
Telomerase from *Daphnia* embryos shows high processivity. A) TRAP assay from egg stage 1 embryos and 1 week old adults of *D*. *pulex* and *D*. *pulicaria*. 250 ng of *Daphnia* extract was used in each lane. B) Quantification of the TRAP assay displayed in 2 A. C) Quantification of the processivity of telomerase from each displayed sample. The p values calculated from student T-tests are as follows: * = 6.15x10^-6^, ** = 5.56x10^-6^, *** = 0.0005,**** = 1.5x10^-5^; # = 0.0002, ## = 0.0002 (n = 4).

In order to compare the relative telomerase activities in embryos and adult organisms we assayed the extracts from embryos and 1 week-old adults of two ecotypes, *D*. *pulex* (RW20) and *D*. *pulicaria* (LakeXVI-clone11). As is shown in Figs 3 and 1 week old *D*. *pulex* (lane 1) shows more telomerase activity than *D*. *pulicaria* of the same age (lane 2). On the other hand, both ecotypes have very comparable levels of telomerase activity at the embryonic stage (lanes 3 and 4). *D*. *pulex* adults at 1 week (lane 1) show some increase in telomerase activity (as measured by total intensity of the bands in entire lane) as compared to embryos (lane 3). In contrast to this, *D*. *pulicaria* adults at 1 week show a significant decrease in total telomerase activity (lane 2) as compared to embryos (lane 4). Fig 3B shows quantification of total telomerase activity in Fig 3A. In addition to determining total telomerase activity based on band intensities, the TRAP assay can also measure the processivity of telomerase, which is the ability of an enzyme to catalyze multiple reactions without releasing the substrate. The greater the processivity, the greater the number of repeats added by the enzyme during the time interval of the assay. This can be visualized as the ladder reaching to higher molecular weights. As seen in Fig 3C, a decline in telomerase processivity is observed from embryo stage to 1 week-old stage of both ecotypes.

### Comparison of telomerase activity from *D*. *pulex* and *D*. *pulicaria* at different ages

**Fig 4:**
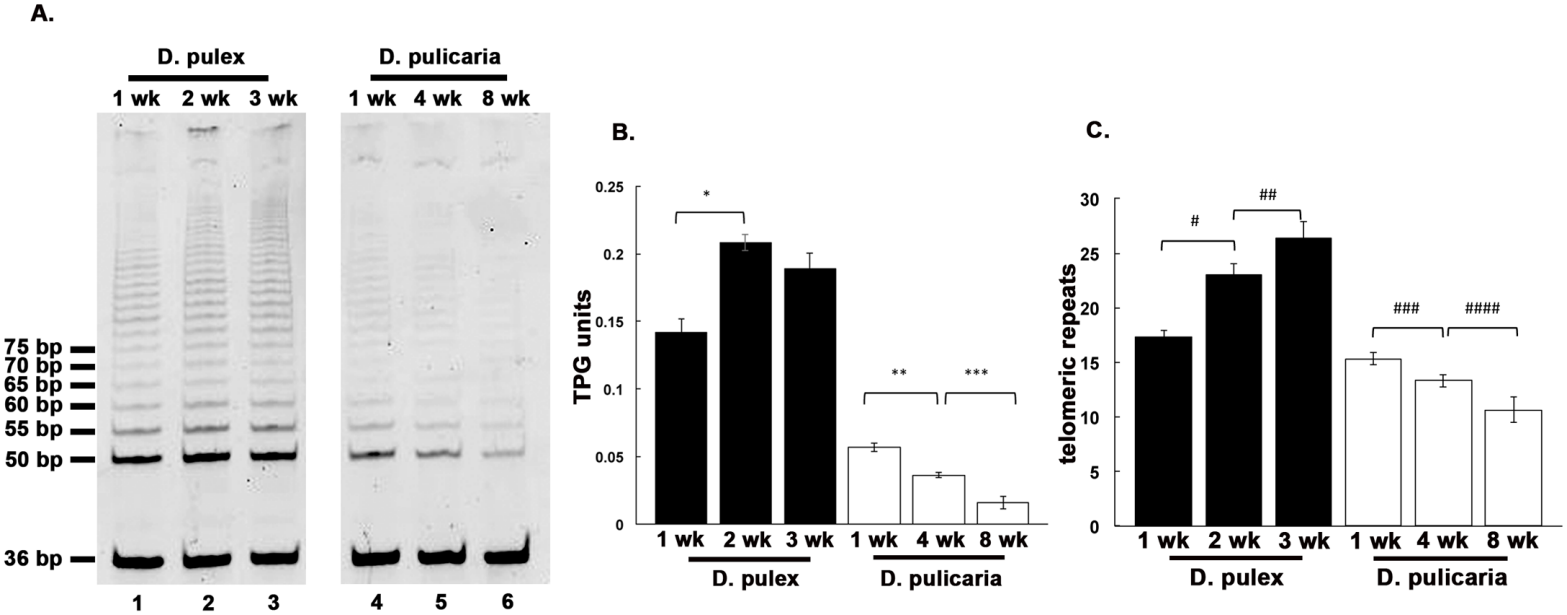
Comparison of telomerase activity at different ages in *D*. *pulex* and *D*. *pulicaria*. A) TRAP assay was performed using *Daphnia* extracts prepared at indicated ages. Lanes 1–3: *D*. *pulex*, lanes 4–6: *D*. *pulicaria*. B) Quantification of the TRAP assay displayed in 4 A. C) Quantification of the processivity of telomerase from each displayed sample. Student T-tests were performed, and p values are as follows * = 0.0005, ** = 0.0006, *** = 0.002, # = 0.001, ## = 0.034, ### = 0.0132, #### = 0.029 (n = 4).

We further investigated the telomerase activity of *D*. *pulex* (RW20 ecotype) and *D*. *pulicaria* (LakeXVI-clone11) at equivalent points in their respective life spans. We performed the TRAP Assay with extracts from 1, 2, and 3 week-old *D*. *pulex* and 1, 4, and 8 week-old *D*. *pulicaria* which were previously determined to correspond to equivalent time points in their respective life spans [24]. As seen in Fig 4A and 4B, *D*. *pulex* (lanes 1–3) showed an increase in telomerase activity from 1 week to 2 week but showed a marginal decline in 3-week-old organisms. In contrast, *D*. *pulicaria* displayed a steady decline of telomerase activity with age, telomerase activity being the highest at 1 week and the lowest at 8 weeks (Fig 4A, lanes 4–6). The telomerase activity was quantified using the Imagequant software and is shown in Fig 4B, which shows a 50% increase in telomerase activity from 1 week-old to 2 week-old organisms in *D*. *pulex*. In contrast, *D*. *pulicaria* showed an age-dependent decline in telomerase activity with about 30% decrease from ages 1 week to 4 week and another 30% decrease from ages 4 week to 8 week. The processivity of the telomerase from these samples was determined and is represented in Fig 4C. In *D*. *pulex*, the processivity of telomerase increased considerably from 1 week to 2 week and showed a small but consistent increase from 2 week to 3 week. In contrast, the processivity of telomerase in *D*. *pulicaria* samples showed a steady decline with age from 1 week to 8 week.

### Telomere length in *D*. *pulex* and *D*. *pulicaria* at various ages

**Fig 5:**
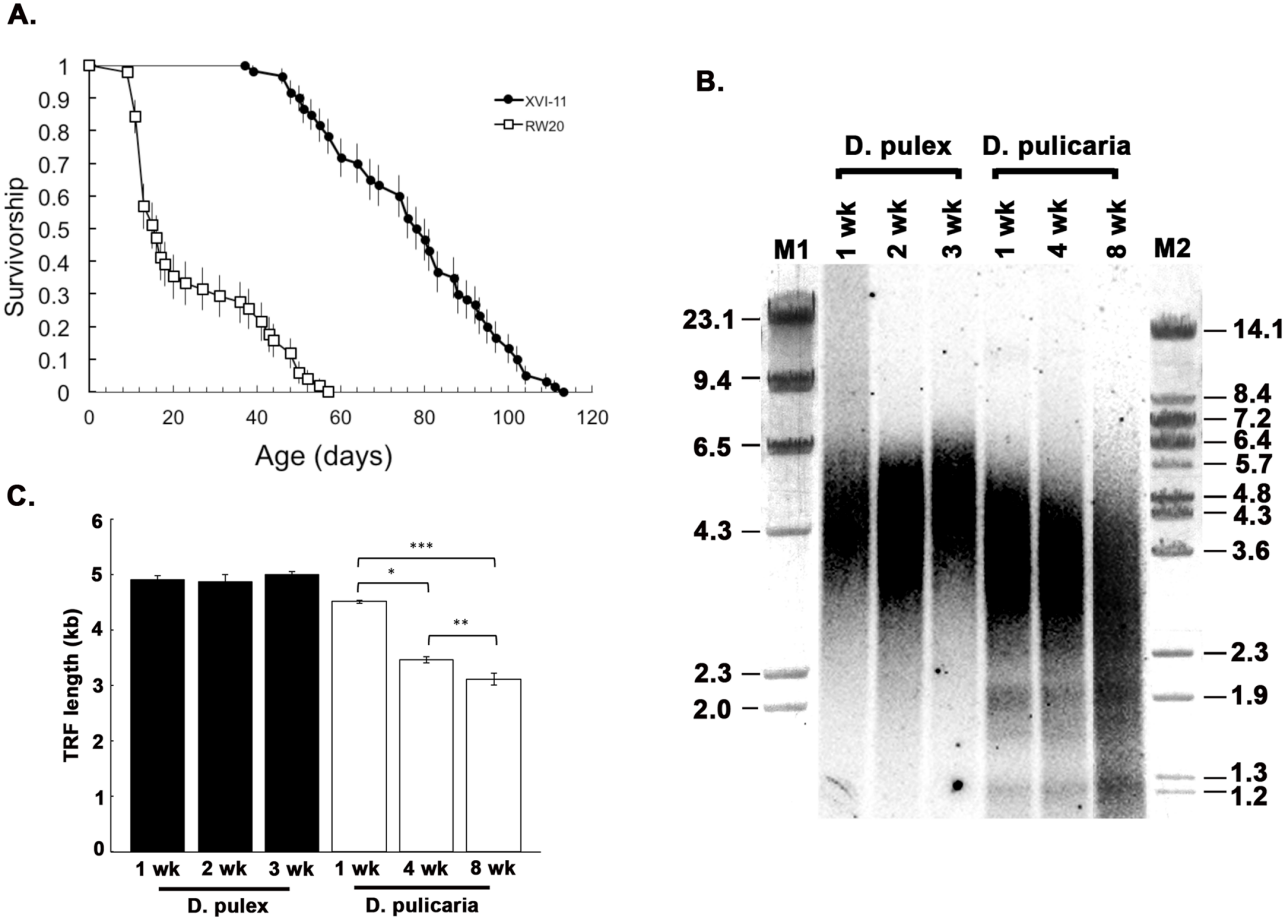
Life spans and telomere lengths in *D*. *pulex* (RW20) and *D*. *pulicaria* (LakeXVI-clone11). A) Survivorship curves of clones RW20 (open squares) and XVI-11 (black circles). Error bars show age-specific standard errors from Kaplan-Meier survival probability estimates. B) Terminal Restriction Fragment (TRF) assay was performed to estimate the average telomere length of the *Daphnia* at various ages. The age in weeks is indicated above each lane. Lanes 1–3: *D*. *pulex*, lanes 4–6: *D*. *pulicaria*. M1 and M2: molecular weight markers; lambda HindIII digest and lambda BstEII digest respectively. C) Quantification of 5 A, error bars indicate standard deviation. Student T-tests were performed and p values are as follows: * = 1.03x10^-5^, ** = 0.007, *** = 2.48x10^-5^ (n = 3).

As shown in Fig 5A, survivorship patterns are substantially different between *D*. *pulex* (clone RW20) and *D*. *pulicaria* (clone Lake XVI-11). RW20 had a median lifespan of 16 d (maximum = 56 d), whereas XVI-11 had a median lifespan of 79 d (maximum = 112 d). A nonparametric log-rank test showed that the survival curves are significantly different (X^2^ = 120.894, *df* = 1, *p* <0.0001). To analyze the average telomere lengths during life span in these two *Daphnia* clones that display markedly different lifespans as well as significantly different telomerase activities (Fig 4B), we performed a terminal restriction fragment (TRF) assay using genomic DNA isolated from 1, 2, and 3 week old *D*. *pulex* and 1, 4, and 8 week old *D*. *pulicaria*. As seen in Fig 5B, the telomere-specific probe detected the TRFs at various ages in both ecotypes. The average telomere length was calculated and is represented in Fig 5B. *D*. *pulex* and *D*. *pulicaria* telomeres at age of 1 week were quite similar in length with *D*. *pulex* at 4.9 kb and *D*. *pulicaria* at 4.5 kb. In *D*. *pulex*, the telomere length did not shorten with age, with the 2 week old and 3 week old telomeres averaging at 4.9 kb and 5.0 kb respectively. In contrast to this, *D*. *pulicaria* telomeres exhibited age-dependent shortening with telomere lengths being 3.5 kb and 3.1 kb at 4 weeks and 8 weeks of age respectively. Therefore, at all equivalent ages, *D*. *pulex’s* average TRF is longer than *D*. *pulicaria’s* average TRF. Thus, the short-lived ecotype *D*. *pulex* displayed no shortening of telomere length with age while the long-lived ecotype *pulicaria* showed an age-dependent decline of telomere length.

## Discussion

Aging is associated with a progressive deterioration of cellular functions leading to a functional decline of organs and tissues. Molecular processes that are considered as characteristics of aged organisms include loss of telomere function, epigenetic genomic changes, declining protein homeostasis, increased cellular senescence, depletion of the stem cell pool, and altered intercellular communication [29]. Telomerase deficiency in humans is associated with premature onset of diseases that are typical of old age [30]. There is evidence of a causal link between telomere loss, cellular senescence and organismal aging that emerged from genetically-modified animal models. Mice with shortened or lengthened telomeres exhibit decreased or increased lifespan, respectively [31,32,33] and aging could be reverted by telomerase activation in telomerase deficient mice [34]. In humans, recent meta-analyses have indicated a strong correlation between short telomeres and mortality risk [35]. Along with its telomere-associated function, telomerase is also involved in DNA damage response and shuttles from the nucleus to the mitochrondria upon oxidative stress to protect the mitochondrial DNA from sustaining oxidative damage [10,13,14,15].

*Daphnia* telomerase protein shows a high degree of homology in the essential functional domains of the protein: the RNA binding domain and the reverse transcriptase domain. *Daphnia* telomeric repeat sequence is TTAGG, a sequence identical to crustaceans *H*. *americanus and G*. *pulex* [28]. Shelterin, a complex formed by six telomere-specific proteins (TRF1, TRF2, TIN2, Rap1, TPP1, and POT1) binds to the telomeric repeats and protects the chromosome ends in mammalian cells [36]. Without the shelterin complex, telomeres are not protected from being recognized by the DNA damage surveillance and are inappropriately processed by DNA repair pathways [36]. Although the shelterin complex is not found in some organisms, there are functional orthologs of the shelterin component proteins. Using the data and tools in PANTHER (a comprehensive, curated database of protein families, trees, subfamilies, and functions) [37] we could identify a *Daphnia* homolog of POT1. POT1-like proteins are present in nearly all eukaryotes [38], and thus it may be of interest in future to identify other proteins that complex with *Daphnia* POT1 since such proteins may be involved in telomere maintenance and chromosome integrity in *Daphnia*.

In our present study, we characterized the telomerase activity, telomerase processivity, and telomere length during the lifespan in two ecotypes of *Daphnia*, the short-lived *D*. *pulex* and the long-lived *D*. *pulicaria*. Our results demonstrate a clear age-associated decline in telomerase activity, telomerase processivity, and telomere length in the long-lived ecotype *D*. *pulicaria*. Surprisingly, the short-lived ecotype *D*. *pulex* showed no decline in telomerase activity and telomere length but an age-dependent increase in processivity of telomerase. The telomere hypothesis of cellular aging states that the telomeres shorten with each cellular replication event until the telomeres are completely eroded and the resulting genomic instability leads to the cellular death in telomerase negative mammalian somatic cells [39,40,41]. Organismal aging, however, is more complex with several different factors in addition to the telomere length and telomerase activity playing a role in the overall lifespan of an organism. Supporting this multifaceted organismal aging process, our results indicate that in the *D*. *pulex* ecotype, telomere length does not decline with age and thus is not the main cause of its short life span.

Overall, our results are in agreement with previous studies in other model organisms with respect to the relationship between the telomerase activity and corresponding telomere length [7,42,43,44]. In *D*. *pulex*, which has high telomerase activity throughout lifespan, there is no decline in overall telomere length. For *D*. *pulicaria*, a decrease in telomerase activity coincides with a decrease in telomere length with age. Comparing telomere lengths of *D*. *pulex* and *D*. *pulicaria* at equivalent points in their lifespan, *D*. *pulex* always has longer telomeres than *D*. *pulicaria* corresponding to higher telomerase activity in *D*. *pulex*. The processivity of *Daphnia* telomerase could be of biological significance in terms its impact on the length of telomeres. Although the telomerase activity may be high in terms of high rate of telomeric repeat addition in TRAP assay, if the enzyme is not processive, the overall telomere length may not be maintained. Thus, in *D*. *pulex* with high telomerase activity and processivity, individual cellular replication events do not effectively erode the telomeres since telomerase can add *de novo* telomeric repeats to the ends of telomeres. However, in *D*. *pulicaria* with telomerase activity and processivity decreasing with age, each cellular replication event leads to shortening of telomeres with age. This is particularly relevant in *Daphnia* as it shows indeterminate overall growth during the entire life span. Although telomerase processivity as a function of aging has not been explored in the past, several different factors are known to contribute to telomerase processivity. These include telomerase RNA template structure, telomere structure, and various proteins that stabilize telomeres [45,46,47]. It’s possible that there is a difference in any of these three factors between the two ecotypes of *D*. *pulex* and *D*. *pulicaria*, which could be investigated in future studies. While our study provides an initial characterization of telomerase activity, processivity, and telomere length in *D*. *pulex* and *D*. *pulicaria*, this work utilizes one isolate or clone from each ecotype. Investigating telomerase activity and telomere lengths in other *Daphnia* species and more isolates or clones of *D*. *pulex* and *D*. *pulicaria* will be of value.

Although much work has been done investigating cellular aging and telomere length, the connection between telomere length and organismal longevity is not straightforward [7,42,43,44]. Formulated by Harley *et al*, the telomere hypothesis of cellular aging postulated that telomeres serve as an internal mitotic clock in telomerase negative mammalian somatic cells and when telomere length is exhausted cellular senescence and the eventual death ensues [39,40,41,48]. Multiple studies have found varying results in terms of telomere length and overall organismal longevity [7,42,44]. Studies involving *C*. *elegans*, a well-established model for molecular biology of aging, have demonstrated that overall telomere length does not affect or predict the organismal longevity [7]. *Danio rerio* shows high levels of telomerase activity throughout its lifespan and maintains telomere length even into late stages of life [44]. No telomere shortening with increasing age is seen in wild-derived mouse strains, as well as in the marine bird *Oceanodroma leucorhoa* [42,49]. The crustacean, *Homarus americanus* (lobster), was investigated to determine overall telomere length and telomerase activity during aging. These organisms display such extensive lifespans that some predict would be nearly immortal if provided with optimal environment [50,51]. Lobsters display telomerase activity throughout their lifespan with a relatively unchanged telomere length [28]. Although cellular aging may be well defined by the telomere hypothesis of cellular aging, organismal aging is a multifactor process with a more complicated relationship between telomere length and organismal longevity [7,42,44,49].

The above results lead to the question that if telomere erosion and hence genomic instability is not the cause of the characteristic short life span of *D*. *pulex* what may be causing this remarkably short life span in *D*. *pulex*? Organismal aging is a multifaceted process and the ability to respond to and survive the proteotoxic stress is an important factor in determining the longevity of an organism [52,53]. There are several theories of aging; however, studies have shown that the pathologies and phenotypes associated with aging may stem from damaged proteins and the inability to repair or eliminate these damaged molecules from cells [52,53,54]. Our previous work has established that the induction of chaperone HSP70 in response to heat shock in *D*. *pulex* declines rapidly with age making it highly susceptible to proteotoxicity. In contrast, *D*. *pulicaria* continues to show a robust heat shock response and chaperone HSP70 induction past the midpoint in its life span, thus enabling it to survive proteotoxicity [24]. Although there are many different aspects of the aging process, it is possible that the ability to respond to proteotoxic stress is a better determinant of organismal life span instead of overall length of the telomeres. In other words, the cellular damage arising from being unable to respond appropriately to proteotoxic stress may lead to death before the telomere length declines. In addition to heat stress, oxidative damage to proteins also causes proteotoxicity and in multiple organisms investigated, better ability to respond to oxidative stress is correlated with longer lifespans [29]. In the marine crustacean, *Acartia tonsa*, oxidative damage was found to be greater in the shorter lived male individuals of the species indicating that oxidative damage may play a role in the overall lifespan of this crustacean [55]. A study done in honeybees identified vitellogenin as a protein that protects the organism from oxidative stress and contributes to longevity [56]. *Daphnia* contain a vitellogenin homolog that could be investigated for any differences in *D*. *pulex* vs *D*. *pulicaria* for its role in protection from oxidative damage [17]. As *Daphnia* is emerging as a model system in aging studies our results reveal a non-concordance between telomerase activity, telomere length and the overall organismal aging and indicate that factors other than telomere length maintenance contribute to strikingly short life span in *D*. *pulex*.
